# Direct identification of *de novo* mobile element insertions from single molecule sequencing of human sperm

**DOI:** 10.1101/2025.10.25.684559

**Published:** 2025-10-26

**Authors:** Stacy Li, Kenneth Aston, Joseph G Gleeson, Aaron Quinlan, Xiaoyu Yang, Peter H Sudmant

**Affiliations:** 1. Department of Integrative Biology, University of California - Berkeley; 2. Center for Computational Biology, University of California - Berkeley; 3. Division of Urology, University of Utah Health; 4. Department of Human Genetics, University of Utah; 5. Department of Biomedical Informatics, University of Utah; 6. Department of Neurosciences, University of California - San Diego

## Abstract

Mobile element insertions (MEIs) are a significant source of human genetic variation, yet the rates and properties of *de novo* MEIs are poorly characterized due to technical limitations in sequencing technology. Here, we directly sequenced individual gametes from sperm samples of 14 donors (aged 28–62) using highly accurate PacBio long-read sequencing to identify *de novo* retrotransposition events without familial inference. We developed a “self-alignment” strategy using personal genome assemblies that enables high-precision, single-read detection of *de novo* MEIs. Using this method, we identified 51 *de novo* Alu insertions, revealing 7-fold variation in Alu retrotransposition rates between individuals (0.023 to 0.17 insertions/gamete). We found a significant increase in Alu activity with paternal age, yielding an additional 0.003 insertions/gamete/year, of additional paternal age, representing a direct observation of age-associated increases in structural variant (SV) mutation rates. These active Alu elements are predominantly comprised of the evolutionarily young AluYa5 and AluYb8 subfamilies and bear characteristic molecular signatures of target-primed reverse transcription (TPRT). Our population-averaged rate of 7.4 insertions per 100 gametes aligns well with previous population genetic estimates, validating both direct observation and population approaches for estimating *de novo* MEI rates. These results establish direct gamete sequencing as a powerful method for characterizing germline mutation processes and reveal age as a significant determinant of *de novo* retrotransposition in the male germline.

## Introduction

*De novo* mutations (DNMs) originate from inaccuracies that occur during parental gametogenesis. These mutations, arising from errors in DNA replication, repair, and recombination, range in size from single base pair substitutions to megabase-scale rearrangements and aneuploidies. DNMs are not uniformly distributed throughout the genome: clusters of DNMs occur more frequently at recombination hotspots, and recurrent genic DNMs underlie multiple neurodevelopmental disorders (Wilfert et al. 2017; Francioli et al. 2015; Hinch, Donnelly, and Hinch 2023). Additionally, the number of genome-wide single nucleotide variant (SNV) DNMs steadily increases with parental age. Phasing of DNMs reveals that the majority of SNV DNMs originate from the paternal gamete. Together, this *paternal age effect* yields an increase of 2 additional *de novo* single nucleotide variants (SNVs) per year of paternal age at conception. Even correcting for the increased number of meioses incurred through the male reproductive lifespan, the male germline mutation rate still increases with age (Kong et al. 2012; Jónsson et al. 2017; Goldmann, Veltman, and Gilissen 2019). Comparative analysis of DNMs across amniotes attributes the paternal mutation bias to differences in endogenous DNA damage and DNA repair response, which are further magnified in aging (de Manuel, Wu, and Przeworski 2022; Kong et al. 2012).

Estimates of DNM rates have almost exclusively been derived from short-read whole-genome sequencing of small variants, leaving a gap in our understanding of *de novo* structural variants (SVs). Short-read analysis of more than 2000 trios with autism spectrum disorder (ASD) found a significant increase in dnSVs in probands compared to unaffected siblings attributed to mutations borne by the paternal gamete (Belyeu et al. 2021). However, short reads often fail to capture larger *structural variants* (SVs) and variants occurring in highly identical or repetitive regions of the genome (e.g. segmental duplications, centromeres). As a result, the true rate of *de novo* SVs (dnSVs) is systematically underestimated despite their relevance in human disease.

Long-read sequencing improves both recall and sensitivity of SV discovery, vastly expanding the landscape of genetic variation that can be assayed. For example, DNM discovery in family studies is significantly improved by long-read data, increasing the *de novo* SV yield by over 30% (Noyes et al. 2022). More recently, analyses of T2T genomes identified *de novo* mutations in several previously inaccessible regions of the genome including STRs, centromeres and the Y chromosome (Porubsky et al. 2025). However, family studies can only assess inherited mutations which are compatible with life. Furthermore, family studies are inherently limited by the number of offspring assessed, with each individual representing the product of only two independent parental meioses.

Transposable elements (TEs) are repetitive sequences capable of moving throughout the genome. Retrotransposons, a subclass of TEs, comprise over 25% of the human genome (Hoyt et al. 2022). Retrotransposons propagate via reverse transcription and integration into a new locus in the genome, a process termed *target-primed reverse transcription* (TPRT). Amplification and mutation of retrotransposons underlie both local-scale instability and long-range evolutionary innovations in genome sequence and structure (J. A. Bailey, Liu, and Eichler 2003). Modern TE activity in humans is limited to three subclasses of retrotransposons: Alu, LINE-1 (L1), and SVA elements (Cordaux and Batzer 2009). These subclasses, listed in order of estimated activity, can be further divided into subfamilies on the basis of sequence identity and phylogenetic age estimates. Most ancient sequences have diverged and become retrotransposition-incompetent (i.e. *dead*), whereas younger copies are frequently polymorphic and linked to increased risks of disease and genomic instability (Payer et al. 2017; Nazaryan-Petersen et al. 2016). Only a fraction of young elements, known as *source elements*, remain active in the human genome (Cordaux, Hedges, and Batzer 2004; Hoyt et al. 2022; Mills et al. 2007). The majority of *de novo* retrotransposition events occur in the male germline (Jerzy Jurka et al. 2002). Furthermore, mobilization of source elements in the germline is associated with both rare and recurrent genetic disease (Hancks and Kazazian 2012). Together, this class of genetic variation is important for both human disease and evolution.

Here, we sought to directly estimate the properties and rates of *de novo* Alu element insertions in the male germline by using highly accurate long-read sequencing to directly sequence gametes. Alu elements are an active and medically relevant class of mutation, estimated to occur in approximately 1 out of every 20 to 100 live births (Konkel et al. 2015; Feusier et al. 2019; Werling et al. 2018; Cordaux et al. 2006; Xing et al. 2009; Deininger and Batzer 1999). As a result of their relatively low frequency, *de novo* Alu activity is challenging to study through conventional pedigree-based approaches: for example, recent analyses of a four generation pedigree identified just a single *de novo* SVA element insertion and no Alu insertions(Porubsky et al. 2025).

We introduce a *self-alignment* (i.e. personalized genome-based) strategy that improves variant calling precision by reducing reference bias through the use of sample-specific genome assemblies. This approach enables reliable detection of *de novo* events supported by single reads, facilitating direct observation of active mutagenic processes in the germline.

## Results

### A framework to identify de novo retrotransposition events in single reads

Conventional germline variant callers rely upon read-depth and split-read approaches to identify variants. However, these approaches are less suitable for non-germline variants, such as somatic and *de novo* variants. These variants occur at low allelic fractions (AF), making them challenging to distinguish from sequencing error, especially in samples where allelic heterogeneity is expected (e.g. tumors). Numerous strategies are employed to optimize sensitivity and specificity in detection of low-AF variants. One common approach reduces erroneous calls by comparing candidate variants against a reference set of variants (e.g. matched tumor-normal variant calling). Another common approach, ensemble variant calling, identifies high-confidence variants by intersecting multiple callsets. Inspired by these techniques, we developed a novel approach for directly identifying and phasing *de novo* variants in gametes without a parental reference.

**Figure 1:**
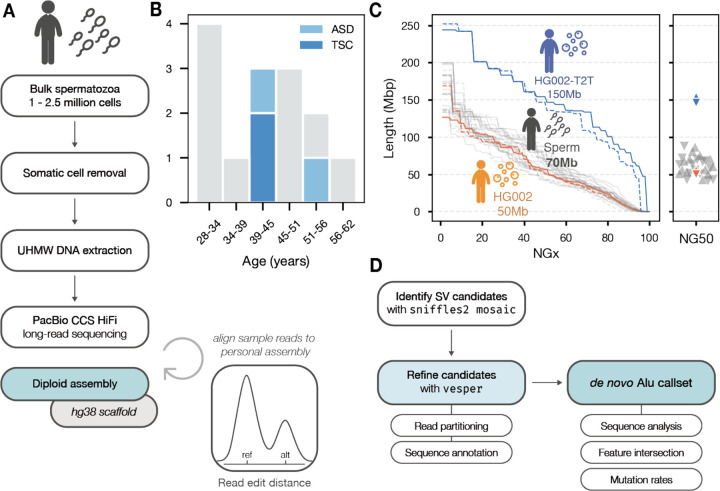
Identifying de novo retrotransposition events from individual sperm cells. Experimental design and assembly quality metrics. **A)** Workflow for DNA extraction, sequencing, genome assembly, and self-alignment. **B)** Histogram of donor age. **C)** Assembly contiguity comparison between sperm-derived assemblies and reference datasets, showing comparable N50 values. **D)** Variant calling pipeline using self-assembly alignment and ensemble methods with vesper refinement.

We used highly accurate PacBio HiFi long-read sequencing on bulk sperm samples from 14 individual donors, including four samples from donors with children affected by TSC (tuberous sclerosis complex) or ASD (autism spectrum disorder) (Fig. 1A–B). We sequenced each sample to high depth, capturing between 1 million and 2.5 million unique cells per individual. We estimate that the vast majority of cells are unique products of a single meiosis and covered by at most 4 reads per cell (see Methods).

Next, we created phased diploid assemblies for each individual using *hifiasm* (Cheng et al. 2021), yielding an average NG50 of ~70 Mb per haplotype. The majority of sperm-derived haplotype-resolved assemblies were comparable in contiguity and quality to an assembly generated from a diploid control dataset (HG002 HiFi reads from HPRC, B-lymphocyte) (Liao et al. 2023) (Fig. 1C). We then aligned reads from each sample to its matched genome assembly (“self-alignment”). This “self-alignment” approach increases precision by minimizing the edit distance between non-SV bearing reads and the reference genome, enabling us to distinguish true *de novo* events from alignment artifacts and allelic variation present in the donor individual.

Following alignment, we performed variant calling in two phases (Fig. 1D) (see Methods for full details). Briefly, variant candidates were called using *sniffles2* (Smolka et al. 2024) using the low-frequency “mosaic” variant detection mode, optimizing for sensitive detection of variants occurring in single reads by removing default quality control filters. In the second phase, insertion sequences were annotated using RepeatMasker to identify candidate retrotransposition events (N. Chen 2004). These candidates were then subjected to stringent filtering by variant quality and individual support read quality, optimizing for variant precision (see Methods). To facilitate this process, we developed a toolkit called *vesper* to automatically annotate insertion sequences and assess read quality.

### Self-assembly alignment improves variant calling precision

We developed a simulation framework to benchmark our self-alignment approach against reference-based alignment, called *nova*. This simulator introduces predefined variants to individual reads from a reference dataset.

**Figure 2:**
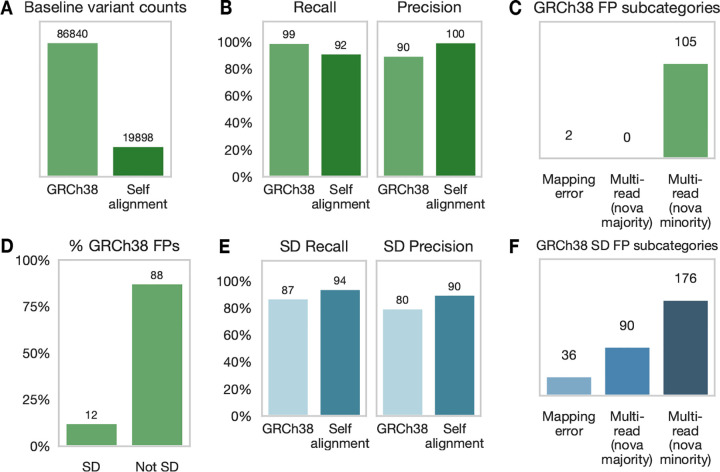
Simulation and detection of synthetic single read Alu insertions. *nova* simulates *de novo* Alu insertions by sampling and modifying reads from a dataset of long reads. **A**) Baseline variant counts comparing GRCh38 vs self-alignment approaches. Low-frequency variant calling with *sniffles2 mosaic* produces an excess of variant candidates. Greater sequence similarity between reads and personal assembly in self-alignment reduces the number of variant candidates. **B**) Recall and precision metrics for *nova-*simulated Alu insertions. GRCh38 alignment recalls 99% of *nova* reads, but incorporates them into more false positive calls. Self-alignment is highly precise, but at the cost of imperfect recall. **C**) Categorization of false positives produced by *sniffles2 mosaic* after GRCh38 alignment. Mapping error refers to variants incorporating single mismapped *nova* reads. Multi-read (*nova* majority) variants incorporate multiple *nova* reads, suggesting clustering of *nova* reads into an imprecise and possibly chimeric Alu call. Multi-read (*nova* minority) variants are chimeric variants that incorporate both *nova* reads and reads supporting germline variants.

First, we established a baseline callset for each strategy by performing variant calling on the HG002 HiFi dataset without addition of the synthetic reads. We performed alignment with two strategies: (1) a standard approach with alignment to the GRCh38 reference genome, and (2) a self-alignment approach to the T2T-HG002 diploid genome assembly (Jarvis et al. 2022). Variant calling with self-alignment yielded 78% fewer variants compared to the standard GRCh38 alignment strategy (Fig. 2A).

Next, to confirm that mapping to GRC38 led to an excess of false positives we used *nova* to generate synthetic single-read *de novo* Alu insertions at random positions throughout the genome and performed variant calling in the presence of these synthetic reads (*nova* reads). We defined true positives (TPs) as variants of any filter status, supported by single *nova* reads mapped to the same position as the original unmodified read. Similarly, we defined false negatives (FNs) as single *nova* reads that were not identified in the callset. Lastly, we defined false positives (FPs) as variants of any filter status, containing single mismapped *nova* reads or incorporating more than one *nova* read per variant or additional non-*nova* reads.

Our initial whole-genome simulations confirmed that self-alignment yields near-perfect precision at a slight cost to recall (GRC38 99% recall, self-alignment 92% recall, Fig 2B). More importantly, self-alignment eliminated all false positives, achieving 100% precision compared to 90% for GRCh38 alignment (Fig 2B). Analysis of reads supporting GRCh38 FPs revealed that the majority of FPs were multi-read “chimera” variants supported by a mixture of germline variant reads and *nova* reads (Fig 2C). Regional stratification of GRCh38 FPs also revealed an almost 2-fold overrepresentation of FPs in segmental duplications (12%) compared to the proportion of the genome occupied by segmental duplications (7%) (Fig. 2D) (Jeong et al. 2025). Segmental duplications are known to be enriched for ancient Alu sequences, which are both a cause and consequence of Alu-Alu mediated recombination and expansion of segmental duplication. (J. A. Bailey, Liu, and Eichler 2003).

We further assessed self-alignment performance via simulations exclusively targeting segmental duplications. We found that self-alignment improved both recall and precision in segmental duplications (Fig. 2E). Further analysis of GRCh38 FPs identifies a majority of “chimera” variants comprising mixtures of both simulated *nova* reads and real reads (Fig. 2F). The remaining FPs resulted from misalignment of reads to incorrect segments in the presence of the synthetic Alu insertion sequence. In comparison, all self-alignment FPs were caused by variants that incorrectly aggregated multiple *nova* reads into a single variant call (Supplementary Fig. 1). Finally, we assessed the performance of our variant filtering approach in reduction of self-alignment FPs. 91% of FPs were eliminated after filtering, confirming the appropriate application of filters to exclude incorrect variants (see Methods). Together, these results demonstrate that de novo Alu insertions can be accurately called from single reads mapped to a self-assembly.

### Direct identification of *de novo* mobile element insertions from long reads

**Figure 3:**
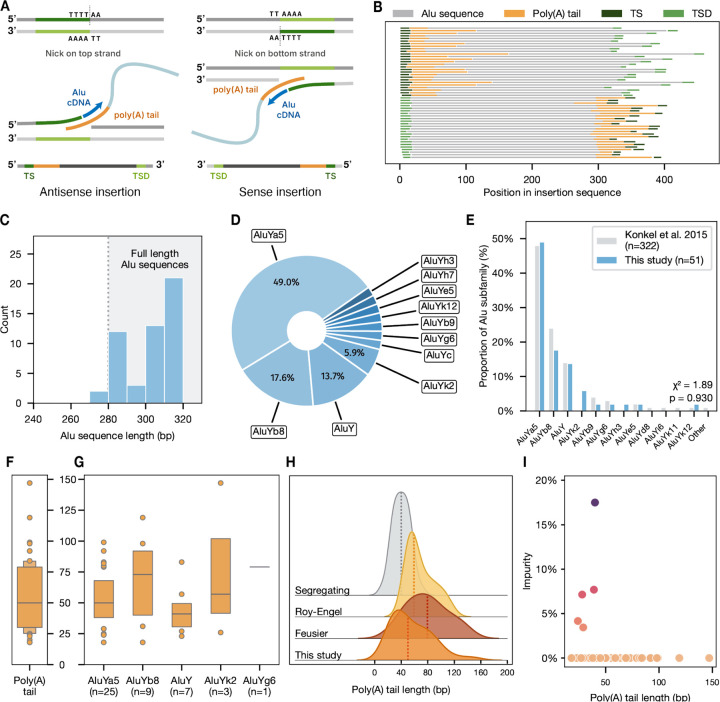
Molecular features of de novo Alu insertions. **A)** An illustration of target-primed reverse transcription of active Alu elements. ORF2p cleaves the top or bottom strand at a 5’-TT/AAAA-3’ motif. Cleavage of the “top” strand results in an *antisense* Alu, characterized by a duplication of the target site region on the 5’ end of the top strand. Cleavage of the “bottom” strand yields a *sense* Alu and duplication of the target site region on the 5’ end of the bottom strand. **B)** Graphical representation of discovered *de novo* Alu insertions and their characteristic features. Alus are partitioned by their orientations (antisense, sense). The target site region and TSD are depicted in different shades of green to distinguish the progenitor sequence from the duplicated sequence. **C)** Histogram of Alu sequence lengths identified by RepeatMasker. The interval highlighted in gray indicates the range of full length Alu sequences. Variation in full length sequences stems from poly(A) tail length beyond the stretch represented by the Dfam consensus sequences. **D)** Subfamily composition of *de novo* Alus. All identified Alus are members of the AluY subfamily and its derivatives. AluYa5 and AluYb8 are disproportionately active compared to other subfamilies, including AluY. **E)** Comparison of *de novo* Alus and segregating Alus analyzed in the 1000 Genomes Project. Segregating Alus originate from *de novo* Alu mobilization events in the recent evolutionary past. The distribution of subfamilies represented in the 1000 Genomes data closely matches the distribution of *de novo* insertions in our study. **F, G)** Boxenplot distribution of *de novo* poly(A) tail lengths in total and across subfamilies. The median length (50 nt) is marked by the center line dividing the upper and lower quantiles. Outliers are shown in points above and below the range of the quantile boxes. **H)** Kernel density estimation plot of *de novo* Alu poly(A) tail lengths in this study compared to two previous studies (Roy-Engel et. al 2005: n = 16; Feusier et al. 2019: n = 11). The dotted lines represent the median poly(A) tail lengths for each category (from top to bottom: 30 nt, 48 nt, 67 nt, 50 nt). **I)** Pol(A) tail purity.

We next applied our approach to identify *de novo* Alu retrotransposition events from individual sperm samples. Alu retrotransposition depends on the activity of ORF2p, a combined endonuclease and reverse transcriptase encoded by autonomous L1 retrotransposons (Feng et al. 1996; Dewannieux, Esnault, and Heidmann 2003). ORF2p operates as part of a ribonucleoprotein complex, binding ORF1p (a RNA chaperone, co-translated with ORF2p) and a single compatible transcript. ORF2p nicks genomic DNA at a 5’-TT/AAAA-3’ motif (J. Jurka 1997) (Fig. 3A). Cleavage by ORF2p exposes a single-stranded 3’ poly(T) sequence used to prime reverse transcription from the poly(A) tail of the Alu transcript, followed by a second strand nick to prime second-strand synthesis and integration of the template Alu into the genome. This process, called *target-primed reverse transcription* (TPRT), frequently results in target site duplications (TSDs) at the 3’ end of the insertion breakpoint (Beck et al. 2010; Batzer and Deininger 2002).

We identified a total of 51 high-confidence full-length *de novo* Alu insertions in single long reads, capturing both the Alu sequence and the genomic context of the insertion (Table 1). We identified characteristic features of each Alu, including the insertion orientation, poly(A) tail, target site (TS) and TSD for each insertion (Fig. 3B). We find approximately equal numbers of sense and antisense oriented Alu insertions, reflecting genome-wide proportions of Alu orientation (Cook et al. 2011). We also identified a single, full length LINE1 insertion on chr3, which contains a tranduced sequence from an active element 25 Mb away. For the remainder of the manuscript however we focus on the properties of the Alu insertions.

The majority of identified sequences are full length, spanning the main body of the Alu sequence (280bp) and terminating in a poly(A) tail of variable length, with the exception of a small minority (7.8%, 4/51) of sequences with 5’ truncation ranging from 5 to 35 bp (Fig. 3C). This proportion is similar to the 14% of 5’ truncated Alus identified in population-scale sequencing of segregating Alus in the 1000 Genomes Project (p = 0.06; two-proportion z-test) (Konkel et al. 2015).

### Segregating Alu subfamilies are representative of *de novo* Alu subfamilies

Mutations accumulated in Alu source elements are inherited by descendant copies, resulting in Alu lineages with variable sequence content and activity. Phylogenetic analysis of these elements partitions human-specific Alus into three major groups: the evolutionarily ancient Alu and AluS subfamilies, and the more recent AluY subfamily (Liu et al. 2009; Batzer and Deininger 2002). Analysis of segregating Alus suggests that only AluY-derived elements remain active in the human genome (Konkel et al. 2015). Consistent with this, we find that all 51 of our *de novo* Alu elements belong to AluY and its derivative subfamilies, with AluYa5 and AluYb8 together comprising over 60% of identified insertions (Fig. 3D). The distribution of active AluY subfamilies in the germline is also remarkably consistent with the population genetically identified Alu profile established by the 1000 Genomes Project (p = 0.936: chi-square goodness of fit) (Fig. 3E) (Konkel et al. 2015). AluYk2 (n=3), a recent addition to the Dfam repeat database absent in the 1000 Genomes analysis, appears to mobilize at substantially lower frequencies than the highly proliferative AluYa5 despite similar copy numbers in the human genome (AluYk2: n=4105; AluYa5: n=4408, GRCh38) (Fernandes et al. 2020).

### Sequence features of *de novo* Alu insertions

#### De novo Alu insertions are marked by significantly elongated poly(A) tails

Experimental dissection of Alu activity has identified a direct association between the length of the poly(A) tract of the RNA and transposition frequency (Dewannieux and Heidmann 2005). Active Alu sequences require a poly(A) tail of at least 10–15 nt to mediate cDNA synthesis during TPRT, with each additional 10 nt contributing to a logarithmic increase in activity, plateauing beyond 50 nt. Furthermore, following integration into the genome, progressive degradation of poly(A) length and sequence content occurs leading to eventual inactivation of Alu elements (Dewannieux and Heidmann 2005; Roy-Engel et al. 2002). This is supported by the observation of relatively shorter A-tails in ancient, immobilized Alus (average: 21 nt) compared to those found in relatively new, segregating Alus (average: 29 nt) (Konkel et al. 2015; Roy-Engel et al. 2002).

We leveraged the extended context of each supporting read to characterize the poly(A) tail length and content of each Alu. We identify a wide distribution of poly(A) tail lengths strongly skewed towards extended poly(A) tracts (min: 18 nt, median: 50 nt, max: 147 nt) (Fig. 3F–G). We find a highly significant difference between segregating and *de novo* A-tail lengths, skewed longer in *de novo* insertions (p < 0.001; Mann-Whitney U test). Two previous studies utilizing Sanger sequencing and short-read sequencing identified similar A-tail length distributions in pathogenic *de novo* Alus, as well as a similarly significant difference between segregating and *de novo* A-tails (Fig. 3H) (Dewannieux and Heidmann 2005; Roy-Engel et al. 2002; Feusier et al. 2019). We observe a greater maximum tail length but no significant difference in median lengths between our study and previous studies of *de novo* events (p = 0.19; Kruskal-Wallis test), highlighting the improved sequence capture afforded by long-read sequencing. *In vitro* studies of tagged Alu sequences and *ex vivo* studies of somatic L1 activity in brain tissues identify similar A-tail lengths, suggesting a shared ORF2p mechanism (e.g. slippage) underlying A-tail expansion (Evrony et al. 2015; Grandi, Rosser, and An 2013).

Next, we examined the purity of each poly(A) sequence. The majority of A-tails were wholly homopolymeric, consistent with *de novo* insertions generated by active source elements. We observed several notable exceptions bearing unusually high frequencies of non-A bases in below-median tail lengths (Fig. 3I) (Supplementary Table 1). Manual examination revealed single-base impurities interspersed within the poly(A) sequence, rather than contiguous tracts of non-A bases. Notably, within individual sequences, all impurities consisted of the same nucleotide (e.g., all T or all G). These impurities were not randomly distributed but instead organized into structured repeat motifs, including direct repeats, inverted repeats, and palindromic sequences. For example, one sequence exhibited tandem TAAA repeats flanking a central palindromic element (TAAAAAT), while another displayed alternating direct and inverted GAAA motifs. This organization suggests non-random mechanisms involved in retrotransposition, potentially involving slippage or secondary structure formation, similar to those responsible for the mutability of similar microsatellite tracts derived from L1 and Alu A-tails (Evrony et al. 2015; Grandi, Rosser, and An 2013). Together, our analysis supports a model in which poly(A) tail length increases upon transcription of an active element, then shortens and degenerates progressively after insertion.

#### Target sites and target site duplications

**Figure 4:**
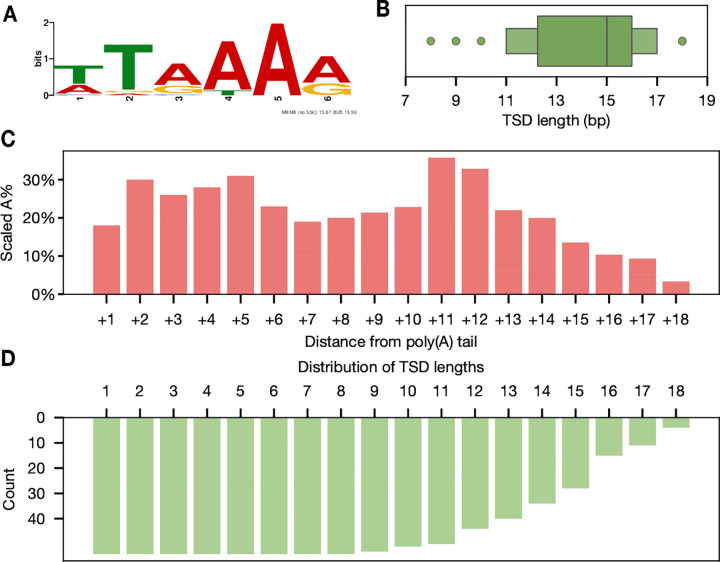
Target site preferences of *de novo* Alus. A) Canonical 5’-TT/AAAA-3’ ORF2p endonuclease motif identified at insertion junctions. ORF2p creates a single strand nick at the 3’-A/T-5’ junction of the complementary strand (see Fig. 3A for illustration). B) Boxenplot of TSD length distribution. We identified perfect TSDs between 8 and 18 nt in length. The median TSD length is marked by the center line dividing the upper and lower quantiles. C) Analysis of adenosine frequency at TSD positions. D) Distribution of TSD lengths. Each bar represents the count of TSDs extending to the given position on the x axis. The number of TSDs decreases after 10 nt.

We analyzed the sequence context at insertion breakpoints to identify motifs associated with Alu insertions. In the immediate context surrounding the breakpoints, we identified the canonical 5’-TT/AAAA-3’ ORF2p endonuclease activity motif (E-value: 1.5 × 10^−4^, Fig. 4A).

ORF2p-mediated TPRT yields TSDs between 8 and 18 nt that are enriched in adenosine content at the proximal positions of the insertion motif region (Szak et al. 2002). We identified TSDs between 8–18 nt (median: 15 nt) in length in all 51 Alu insertions, consistent with shared activity of ORF2p (Fig. 4B, Table 1). Each TSD was identical in sequence between the origin (3’) site and duplicated (5’) site, consistent with *de novo* duplications caused by TPRT.

Although the precise factors governing TSD size remain uncertain, recent structural models of ORF2p guided by cryo-electron microscopy (cryo-EM) suggest a relationship between target sequence and DNA repair factors (Thawani et al. 2024; Ghanim et al. 2025). We hypothesized that the nucleotide composition of the distal TSD may play a role in determining TSD length. We analyzed the proportion of nucleotides at each site (Supplementary Fig. 7–8), followed by the frequency of adenosine at each site as a function of TSD length. Remarkably, we discovered a bimodal enrichment of adenosine proximal to the 3’ end of the Alu sequence in each orientation (Fig 4C–D). The first peak, occurring across the proximal 4 bases of the TSD, corresponds to the poly(A) stretch of the ORF2p motif sequence, followed by the second peak at the distal 11–12 bp of the TSD. Intriguingly, the number of TSDs longer than 11nt declines rapidly. *In vivo* fluorescence analysis of ORF2p-DNA interactions during TPRT identify second-strand cleavage sites at +7, +11, and +19 nt (Ghanim et al. 2025). These positions are consistent with our observed minimum and maximum TSD lengths and the adenosine peak at 11–12 nt. Together these results highlight how characterization of *de novo* mobile element insertions provides support for recent mechanistic insights into Alu insertion.

### Sequence preferences of Alu insertions

#### Sequence preferences of de novo Alus

Several studies report clustering of Alu elements in gene-dense, GC-rich regions of the genome (International Human Genome Sequencing Consortium 2001; Gu et al. 2000; Jerzy Jurka et al. 2002; Grover et al. 2004). These biases are strongest for the ancient AluJ and AluS subfamilies, whereas younger AluY elements appear to be relatively randomly distributed throughout the genome (Brookfield 2001; Jerzy Jurka et al. 2004). However, local sequence context may influence preferential integration amongst available ORF2p target sites.

**Figure 5:**
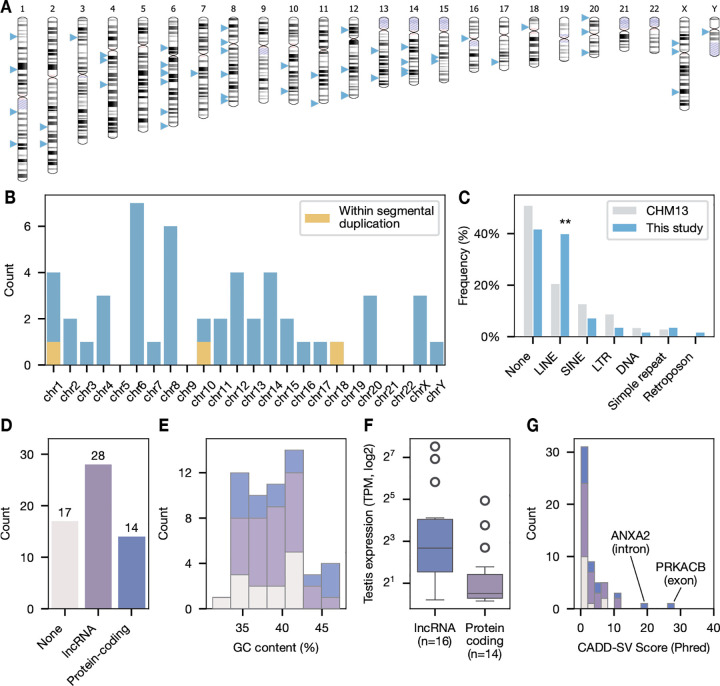
Sequence preferences of *de novo* Alus. **A)** Chromosome ideogram of Alu insertion sites transferred to GRCh38 coordinates. **B)** Histogram of Alu insertions per chromosomes. Three insertions were identified in segmental duplications on chr1, chr10, and chr17. **C)** Distribution of insertion sites absent from (“None”) or covered by repetitive sequence annotations from RepeatMasker. *De novo* Alu insertions are significantly enriched in LINE sequences when compared to the genome-wide proportion (p = 0.008, binomial test with Bonferroni correction). **D)** Distribution of GRCh38 insertion sites covered by GENCODE v48 annotations. 73% (37/51) of insertions intersected with at least one annotated feature. **E)** GC content in the 10 kb window around insertion sites, subdivided by intersected features. The average GC content is 38% across all insertions, similar to the genome-wide average. **F)** Gene expression from GTEx bulk RNA-seq analysis of testis tissue. All 14 intersected protein-coding genes are expressed in the testis. **G)** CADD-SV Phred-scaled scores for all GRCh38 insertion sites.

We began by projecting insertion coordinates from each personal assembly to the GRCh38 reference genome, excluding one insertion without GRCh38-equivalent coordinates (remaining n=50). Three insertions were identified and manually confirmed to reside within segmental duplications on chr1, chr10, and chr17. We identified no insertions on chr 5, 9, 18, 20, or 21, likely due to our small sample size (Fig 5A, B).

Young Alus preferentially “nest” within Alu and LINE-1 elements, effectively disabling further activity (A. Levy, Schwartz, and Ast 2010). To assess whether our *de novo* insertions follow this reported pattern, we performed *de novo* repeat annotation of each assembly. We identified a significant preference of Alu insertions within LINE elements (p = 0.008331, binomial test with Bonferroni correction) (Hoyt et al. 2022), but not Alu elements (Fig. 5C).

Both ancient and segregating Alus are enriched in gene-dense regions of the genome (Grover et al. 2004). Intronic Alus, comprising the majority of genic Alus, have been observed to alter gene expression and function, while rarer exonic insertions are especially likely to yield genetic disease (Wallace et al. 1991; Oldridge et al. 1999; Hancks and Kazazian 2012; Deininger and Batzer 1999; Payer et al. 2017).

We then identified all GENCODE features intersected by insertion sites. The majority (37/50) of Alus intersected at least one feature, with 38% (14/37) of insertions intersecting protein-coding genes and over half (24/37) intersecting at least one long non-coding RNA gene (lncRNA) (Fig. 5D). Nearly all (13/14) of protein-coding gene insertions occurred within introns, reflecting the minor fraction of gene content occupied by exon sequences (Francis and Wörheide 2017). We further assessed the GC content of the immediate 10kb window surrounding each insertion site, finding no significant difference between the average GC% of insertion sites (38%) compared to the genome-wide average of 41% (Fig. 5E). These results support a model in which Alu integration is primarily driven by target site availability and average genome composition rather than specific contextual preferences.

Biophysical simulations of chromatin accessibility suggest that retrotransposons preferentially integrate in open, sterically permissive chromatin regions (Prizak, Gadzekpo, and Hilbert 2025; Michieletto et al. 2019). Under this premise, we conducted an analysis of the GTEx bulk RNA-seq dataset, reasoning that genes expressed in the testis are reasonable proxies of chromatin accessibility. In total, we identified 30/42 intersected genes, including all 14 protein-coding genes (Fig. 5F). 27/37 Alus intersected at least one gene expressed in the testis, with the remaining Alus intersecting lncRNAs with sparse gene expression across all GTEx tissues.

Finally, we used CADD-SV to conduct an exploratory functional analysis of each insertion (Kleinert and Kircher 2022). CADD-SV predicts and scores the effects of SVs using random forest models trained on variants from human and chimpanzee-derived SVs. Scores are Phred-transformed relative to variants of known phenotype from the gnomAD-SV dataset, ranging from 0 (unlikely pathogenic) to 48 (likely pathogenic): for example, a score of 10 corresponds to the 90th percentile of estimated pathogenicity. We scored all 50 variants, including intergenic variants, that were successfully projected to GRCh38 coordinates (Fig. 5G). Out of the five insertions with a score greater than 10, three occurred in protein-coding genes. As expected, the single exonic insertion in *PRKACB* received the highest score of any variant in the dataset. *PRKACB* is one of two catalytic subunits of *PKA*, a critical regulator of human morphogenesis. Germline and mosaic variants of *PRKACB* are associated with congenital malformations and neurodevelopmental disorders (Palencia-Campos et al. 2020). The second highest score corresponds to an intronic insertion in *ANXA2*, a cytosolic RNA-binding protein believed to play a modest role in mediating inflammation through alternative splicing (J. Chen et al. 2022).

### Direct estimation *de novo* Alu retrotransposition rates from gametes

Alu activity in the germline is wholly dependent on the production of ORF2p by LINE-1 source elements, which are typically repressed via hypermethylation and post-transcriptional degradation by the PIWI-piRNA pathway (Gainetdinov et al. 2017; Vagin et al. 2006). However, LINE-1 activity continues to persist at low levels in healthy individuals, yielding both LINE-1 and Alu *de novo* retrotransposition events. Studies in both mouse models and clinical cases of infertility suggest that dysregulation of piRNA biogenesis may result in impaired spermatogenesis and de-repression of LINE-1 elements in early meiosis (Wei et al. 2023; Stallmeyer et al. 2024). We hypothesized that tissue-specific dysregulation of the PIWI-piRNA may occur in the aging germline, akin to decline of transcriptional regulation observed for somatic tissues in aging (Debès et al. 2023; Leote, Lopes, and Beyer 2024; Yamamoto et al. 2022; O. Levy et al. 2020).

**Figure 6:**
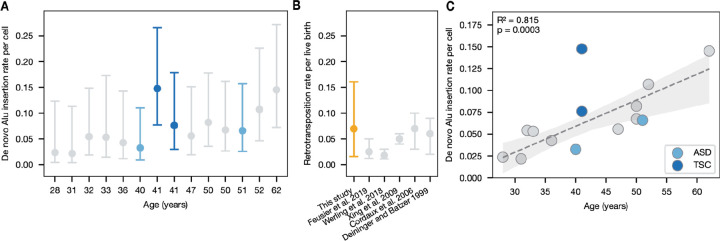
Individual and population-level Alu retrotransposition estimates. **A)** Per-individual de novo Alu retrotransposition rates by age. **B)** Aggregate de novo Alu retrotransposition rate compared to population genetic estimates. **C)** Linear regression of healthy donor rates with 95% CI between age and insertion rate. Individuals with offspring affected by ASD (autism spectrum disorder) and TSC (tuberous sclerosis complex) are highlighted.

We began by calculating the per-sample, per-gamete *de novo* Alu retrotransposition rate as the number of observed Alu insertion events divided by sequencing coverage, as each read represents sampling of a single gamete. (Fig. 6A). Combining our estimates across samples, we estimate an average rate of 7.4 Alu insertions per 100 gametes for all surveyed individuals (95% CI: 5.7–9.6 Alu insertions per 100 gametes). This estimate falls precisely within the distribution of mutation rates derived from pedigree and population studies of Alu activity, suggesting that population estimates of *de novo* Alu activity accurately reflect germline activity (Fig. 6B) (Feusier et al. 2019; Hancks and Kazazian 2012; Hormozdiari et al. 2011).

Next, we examined covariation between age and Alu insertion rates. We observe a 7-fold difference in rates estimated between the youngest and oldest individuals in our dataset, ranging from 0.023 to 0.17 *de novo* Alus per gamete. Notably, we identify a statistically significant increase in Alu retrotransposition with age in healthy samples, estimated at an additional 0.003 Alu insertions per gamete per year of paternal age (p = 0.0003, R^2^ = 0.815, linear regression) (Fig. 6C). As a preliminary exploration of our hypothesis, we used BLAST to search for each Alu insertion sequence and identify high-identity source element candidates in the GRCh38 reference genome. Through manual curation, we found that the majority of candidates were hypermethylated, but identified at least one hypomethylated candidate for each insertion sequence.

Finally, we examined Alu retroposition rates in samples from donors with offspring affected by ASD (n=2, “ASD samples”) and TSC (n=2, “TSC samples”). We did not observe a significant difference in the mutation rates of ASD samples. However, we identify a remarkably elevated mutation rate in one of our TSC samples (p = 0.03, two-tailed z-test). TSC is a neurodevelopmental disease predominantly caused by *de novo* mutations in the *TSC1* and *TSC2* genes. In several cases, TSC has also been shown to be caused by paternal gonadal mosaicism (Verhoef et al. 1999; Uysal and Şahin 2020). However, we found no evidence of Alu insertions nor mosaicism in *TSC1* or *TSC2* in either TSC sample.

## Discussion

This study represents the first successful application of gamete sequencing to characterize *de novo* mobile element mutation processes in the human germline at unprecedented scale and resolution. By optimizing variant calling methods for ultra-low frequency variant detection, we directly isolate *de novo* retrotransposition events without the use of family-based inference.

We demonstrate the feasibility of constructing high-quality genome assemblies from haploid sperm samples. Self-alignment to personal genome assemblies addresses a persistent technical challenge in detecting low-frequency genetic variation: distinguishing true variation from artifacts arising during sequencing and alignment. We developed *nova*, a *de novo* variant simulator that constructs synthetic variants in single reads. Our simulation results demonstrate that self-alignment substantially improves precision compared to standard GRCh38 reference alignment, particularly in complex regions such as segmental duplications. This increase in precision underlies the feasibility of single-read and low allele frequency SV discovery where read depth approaches are not applicable.

We characterize 51 individual *de novo* Alu insertions across 14 individuals, identifying detailed molecular features of each insertion and its genomic sequence context. The subfamily composition of our *de novo* insertions shows remarkable consistency with patterns observed in segregating Alu elements from the 1000 Genomes Project, with AluYa5 and AluYb8 subfamilies comprising the majority of active insertions. This concordance validates both direct observation and population genetic approaches while demonstrating that current retrotransposition activity reflects the same processes that generated segregating variation in recent evolutionary history.

Furthermore, we capture the full span of each element’s poly(A) tail without the use of targeted sequencing or local reassembly. These tails are significantly longer than those observed in ancient, inactive elements, supporting models of progressive tail degradation following insertion. Similarly, analyses of TSD sequence content support structural and mechanistic assessments of ORF2p and TPRT, providing insights into the generation of TSDs of varying size and composition.

We directly estimate *de novo* retrotransposition rates for each individual, providing unprecedented resolution into inter-individual variability in germline mutation rates. We find a significant association between retrotransposition rate and donor age in healthy samples, reflected in the near 7-fold difference between the youngest and oldest donors in our study. We speculate that variation in Alu retrotransposition rates may be a consequence of transcriptional dysregulation in the PIWI-piRNA pathway, resulting in progressive de-silencing of retrotransposons in the male germline. Anecdotally, we observe a significantly elevated mutation rate in a sample provided by a donor with a child affected by tuberous sclerosis complex, suggesting potential associations between individual mutation burden and disease risk that merit further investigation.

Finally, we repeatedly identify close agreement between our direct measurements of retrotransposition rates and estimates derived from population genetic studies. This concordance provides mutual validation of both approaches and suggests that the events we observe in gametes are representative of those that successfully transmit through the germline. This agreement also supports the use of segregating element analysis for understanding historical retrotransposon activity and evolution of retrotransposon lineages.

Our study has several notable limitations. In particular, we rely on statistical estimation of unique reads per cell, precluding direct identification of cell-specific mutations and reconstruction of clonal mutations. Single-cell approaches, while technically more challenging, could provide higher resolution insights into the timing and cellular context of mutagenic events. Additionally, due to the destructive nature of the sampling and sequencing process, we lack the power to follow up on our hypothesis on PIWI-piRNA transcriptional dysfunction.

Overall, we demonstrate that direct sequencing of gametes from even a comparatively small population of individuals has the power to accurately quantify the mutation rate of mobile elements. To our knowledge, we identify the first age-associated increase of any class of *de novo* structural variants. Our results demonstrate the feasibility of using highly accurate long-read sequencing to directly observe germline mutation at the individual level. Future studies utilizing direct gamete sequencing will improve our understanding of structural variants, individual variation in germline mutation rates, and genetic disease risk.

## Methods

### Study design and sample collection

We obtained semen samples from donors from ages 28 to 62, with a median age of 41. Four samples from fathers of children with autism spectrum disorder (ASD, n=2) and tuberous sclerosis complex (TSD, n=2) were shared by the Gleeson lab. All samples were stored at −80°C until processing.

### DNA extraction

Semen samples were thawed on ice and subjected to a gradient separation using 90% PureCeption Isotonic Solution (CooperSurgical Cat. #ART-2100, Cat. #84051010) to remove seminal fluid and contaminating somatic cells. After separation, the cell pellet was washed with 2 mL of PBS and resuspended in 100 uL of PBS in a 2 mL centrifuge tube. DNA extraction was performed using a modified version of the Monarch^®^ HMW DNA Extraction Kit for Cells & Blood (New England Biolabs Cat. #T3050L) extraction protocol. Cell lysis solution (100 uL of Nuclei Prep Buffer, 100 uL of Lysis Buffer, 15 uL of 20 mg/mL Proteinase K, 15 μL of 1M DTT (Goldbio Cat. #DTT)) was prepared and added to each sample, followed by thermal mixing with an Eppendorf ThermoMixer^®^ set to 56°C/300 RPM for 2 hours. RNA digestion was performed by adding 5 uL of 20 mg/mL RNase A and incubating under the same conditions for 20 minutes. The remainder of the DNA binding and wash steps were conducted according to the protocol description. DNA was eluted from glass beads overnight at room temperature using 75 uL of Elution Buffer, followed by centrifugal separation into Eppendorf DNA LoBind^®^ Tubes (Eppendorf Cat. #0030108523). Sample DNA was gently mixed by wide bore pipetting and then allowed to rest for 24–48 hours at room temperature before quantification using the Promega^®^ Quantus^™^ Fluorometer and purity ratio (A260/280, A260/230) assessment with a ThermoFisher NanoDrop Spectrophotometer. Samples were submitted to the UC Berkeley Functional Genomics Laboratory for fragment analysis using an Agilent Femto Pulse system. Samples containing at least 10 μg of DNA with a median fragment length exceeding 50 kB were deemed sufficient for sequencing. DNA was stored at 4°C and shipped on dry ice to the sequencing laboratory.

### Long-read sequencing

Highly accurate long-read whole-genome DNA sequencing was performed at the HudsonAlpha Genome Sequencing Center using the Pacific Biosciences (PacBio) Revio sequencer. We sequenced each sample using two SMRTcells to a target depth of 50X haploid genome coverage with an 18 kb read length, yielding unaligned BAM files for each SMRTcell. Samples with insufficient coverage were resequenced to a maximum of four total SMRTcells. We converted each uBAM to FASTQ format, preserving all tags in the read headers, and used HiFiAdapterFilt to confirm the absence of errant sequencing adapters present in data from previous generation sequencing machines.

### Estimating reads per unique cell

We estimated the maximum number of reads per unique cell using 50X coverage and a normally distributed read size (mean = 18 kb, SD = 2.5 kb), representative of the average values for each sample. Each sample of sperm cells is subsampled from the population of gametes produced by the individual. We assume that this population is large, each meiotic cycle is perfect (produces 4 daughter cells), and all 4 daughter cells are represented in the population. The average sperm cell count and volume in each semen sample is 2 mL of 100 million cells/mL per specimen, for a total of 200 million cells representing approximately 50 million meioses. From this specimen, we sample approximately 2.5 million cells for DNA extraction. Each sample was sequenced with 2 SMRTcells, each requiring 293 ng of DNA input.

To calculate maximum possible per-cell coverage, we assume a perfect yield of 18 kb fragments for each cell,

$$N_{\text{bp}}^{\text{gDNA}}=N_{\text{cells}}\times G=\left(2.5\times 10^{6}\right.\;\text{cells})\times \left(3.2\times 10^{9}\right.\;\text{bp})=8.0\times 10^{15}\;\text{bp}$$

$$N_{\text{fragments}}^{\text{gDNA}}=\frac{N_{\text{bp}}^{\text{gDNA}}}{\mu_{f}}=\frac{\left(2.5\times 10^{6}\;\text{cells}\right)\times \left(3.2\times 10^{9}\;\text{bp}\right)}{18,000\;\text{bp}/\text{fragment}}=4.44\times 10^{11}\;\text{fragments}$$

We then use the mass of DNA loaded for sequencing to calculate the number of prepared fragments for sequencing.


$$N_{\text{bp}}^{\text{library}}=\frac{m_{\text{sample}}}{m_{\text{bp}}}=\frac{586\times 10^{-9}\;\text{g}}{1.096\times 10^{-21}\;\text{g}/\text{bp}}=5.35\times 10^{14}\;\text{bp}$$

$$N_{\text{fragments}}^{\text{library}}=\frac{N_{\text{bp}}^{\text{library}}}{\mu_{f}}=\frac{5.35\times 10^{14}\;\text{bp}}{18,000\;\text{bp}/\text{fragment}}=2.97\times 10^{10}\;\text{fragments}$$

Knowing that our final coverage is 50X, we estimate $N_{\text{reads}}^{\text{sequenced}}$ sequenced from $N_{\text{bp}}^{\text{sequenced}}$:

$$N_{\text{bp}}^{\text{sequenced}}=C\times G=50\times \left(3.2\times 10^{9}\right.\;\text{bp})=1.6\times 10^{11}\;\text{bp}$$

$$N_{\text{reads}}^{\text{sequenced}}=\frac{N_{\text{bp}}^{\text{sequenced}}}{\mu_{f}}=\frac{1.6\times 10^{11}\;\text{bp}}{18,000\;\text{bp}/\text{fragment}}=8.89\times 10^{6}\;\text{reads}$$

Finally, we calculate the maximum number of fragments sequenced per cell:

$${\overset{‾}{N}}_{\text{reads}/\text{cell}}=\frac{N_{\text{reads}}^{\text{sequenced}}}{N_{\text{cells}}}=\frac{8.89\times 10^{6}\;\text{reads}}{2.5\times 10^{6}\;\text{cells}}=3.56\;\text{reads}/\text{cell}$$

### Modeling unique meioses represented per sample

As described in the previous sections, we extracted DNA from approximately 2.5 million cells per specimen, such that the sampling probability per cell is:

$$p=\frac{N_{\text{cells}}}{N_{\text{specimen}}}=\frac{2.5\times 10^{6}\;\text{cells}}{200\times 10^{6}\;\text{cells}}=0.0125.$$

For each meiotic event, the number of its cells appearing in the sample follows a hypergeometric distribution. However, given the large population of cells in the specimen, we can accurately approximate this with a binomial distribution. For a single meiotic event with 4 cells, let X be the number of cells sampled, where:

X~Binomialn=4,p=0.0125;P(X=k)=4kpk(1-p)4-k

We calculate the probability of sampling a single daughter cell per meiosis (“singleton”) as:

P(X=1)=41(0.0125)1(0.9875)3=0.048118

Thus, for 4.81% of all 50 million meioses, we obtain singletons totaling 96.2% of the cells in the sample.


$$N_{\text{singleton}}=N_{\text{meioses}}\times P(X=1)=\left(50\times 10^{6}\;\text{meioses}\right)\times (0.048118)=2,405,900\;\text{singletons}$$

### Analysis

#### Genome assembly

Haplotype-resolved assemblies were generated with hifiasm (v.0.19.5) using the HiFi data for each sample, as well as an additional diploid sample (HG002).


□hifiasm -o {params.prefix} -t {threads} {input}
□


The primary raw assembly graphs (.gfa) for each haplotype were converted to FASTA formatted contigs using gfatools (v.0.5) and assessed using QUAST (v.5.2.0). The average N50 of each assembled haplotype was 70Mb, comparable to the N50 of the HG002 assembly haplotypes. Contigs were scaffolded to the GRCh38 (hg38) reference genome using RagTag (v.2.1.0) and merged into a final FASTA file containing both haplotypes. We used Flagger (v.1.1.0) to detect errors in each assembly and generate a BED file of high-confidence regions across the genome.

#### Sequence alignment

We used minimap2 (v.2.26) to align HiFi reads for each sample against both the sample’s own assembly (“self-assembly”) and the GRCh38 (hg38) reference genome. We used samtools (v.1.21) to filter the output file, retaining only reads with MAPQ ≥ 20.


□minimap2 {params.refgenome} {input.hifi} \
-ax map-hifi -Y -y -L --eqx --cs --MD \
-R ‘{params.readgroup}’ | samtools view \
-q 20 -bT {params.refgenome} -o {output}


□Following alignment, we used deeptools (v.3.5.2) to query read depth, assess read alignment qualities, and calculate genome-wide coverage for each sample ahead of variant calling (Ramírez et al. 2014).

#### Genome annotation

We used liftOver (v.447) to transfer centromere, segmental duplication, and GENCODE v44 annotations from the GRCh38 reference genome to each of our scaffolded assemblies. Chain files for liftOver were generated from the scaffolded PAF file and paf2chain (v.0.1.1, https://github.com/AndreaGuarracino/paf2chain). To ensure the veracity of repetitive sequence annotations, we performed *de novo* annotation of each scaffolded assembly with RepeatMasker (v.4.1.2.p1).

#### Variant calling

We used *sniffles2* (v.2.5.2) in mosaic mode to raise a “first pass” callset of SV candidates. We optimized for recall of *de novo* events by lowering the minimum read support to a single read while also limiting the allele frequency of calls to no more than 0.10. We applied the `--qc-all` option to emit all variants regardless of FILTER flag.


□sniffles --input {input.bam} \
--vcf {output.vcf} \
--reference {params.refgenome} \
--tandem-repeats {params.repeats} \
--threads {threads} --mosaic \
--minsupport {params.minsupport} \
--mapq {params.mapq} \
--output-rnames \
--mosaic-af-min {params.mosaic_af_min} \
--mosaic-af-max {params.mosaic_af_max} \
--mosaic-qc-strand={params.mosaic_qc_strand}


□We obtained support read names by using the --output-rnames option to emit read names in the INFO field of the VCF.

#### Identifying de novo mobile element insertions

We developed *vesper*, a variant refinement tool, to identify individual insertion candidates containing Alu and LINE-1 sequences. vesper comprises two subcommands: annotate and refine.

*vesper annotate* takes a sniffles2 output VCF file as input and returns an modified VCF as output. The primary function of *vesper annotate* is variant sequence analysis and intersection. Insertion sequences are written to a FASTA file and analyzed with RepeatMasker to identify repetitive sequence content. Optionally, genome annotation files (GFF) can be provided for predefined annotation. Entries from each file are read from each file and cached in a SQLite database, enabling rapid annotation of proximal and overlapping genomic features. Both predefined annotations and RepeatMasker annotations are written directly into the INFO field of each variant, maximizing compatibility with other VCF utilities like bcftools.

*vesper refine* takes the output of vesper annotate as input and calculates confidence scores based on read-level evidence and contextual features at the call site, returning a modified VCF as output. The confidence score (floating point value between 0 and 1) is calculated by adjusting a base score of 1 by a quality factor determined by read metrics. For each variant, *vesper* fetches the reads at the variant position (POS), partitioning reads into two groups: “supporting” (identified via the read name(s) denoted in the INFO field) and “non-supporting” reads. It then calculates metrics for each read group, such as average mapping quality and soft-clip percentage, setting the non-supporting reads as a baseline. *vesper* then calibrates a final confidence score scaled by the metrics of the supporting read group. By default, *vesper* automatically assigns a quality score of 0 to variants supported solely by secondary or supplementary alignments.

#### Filtering and detection of de novo Alu events

We used *vesper annotate* and bcftools to identify and filter insertions with Alu annotations. Using bcftools (v.1.19), we pre-filtered the callset to ≤ 10 kb insertion variants on primary autosomes (1–22) and sex chromosomes (X, Y) with between 40 to 80X coverage at the call site. After passing this VCF to *vesper annotate*, we further restricted the callset to ≥ 300 bp Alu-annotated variants with ≤ 3% sequence divergence from the Repbase/Dfam consensus sequence, representing the likeliest *de novo* Alu sequences (Bennett et al. 2008). We used *vesper refine* to further analyze each variant, removing low-confidence variants without primary read support and variants in centromeric regions.

#### Analysis of ORF2p motif, target site, and extended context

We performed the proximal insertion context analysis by extracting the 4 bases surrounding the insertion breakpoints. For sense-oriented Alu insertions, we extracted the 4 base pairs upstream of the “left” TSD start coordinate and concatenated them to the 4 base pairs following the poly(A) tail end coordinate. For antisense-oriented Alu insertions, we extracted the 4 base pairs upstream of the poly(A) tail end coordinate and concatenated them to the 4 base pairs following the “right” TSD end coordinate. For target site analysis, we used the sequences extracted by the target site duplication detection algorithm. We examined extended sequence context by extracting the 20 bases upstream of the “left” TSD and downstream of the “right”. All contexts were extracted from the native support read sequence.

For both analyses, we used MEME (Multiple Em for Motif Elicitation, Version 5.5.8) to analyze the sequence contexts around each insertion breakpoint (T. L. Bailey and Elkan 1994).

#### Genic feature analysis

We used bedtools intersect (v.2.31.1) with liftOver-transferred genome annotation files from GENCODE (v44) to identify genic features present at insertion loci and assess the intron/exon boundaries for each feature relative to our scaffolded assembly coordinates. We used Ensembl gene IDs to query gnomAD (v.4.1.1) LoF o/e values and GTEx (v10) median gene expression (TPM) values for each intersected feature.

## Supplementary Material

1

## Figures and Tables

**Table 1:** ***De novo* Alu insertions identified in human gametes**.

Chromosome	Position	Subfamily	Orientation	TSD length	TSD sequence	Poly(A) tail length	Poly(A) tail impurity (%)
chr1_hap1	32597902	AluY	Sense	18	AAAAAAAATTAGCTGGGC	42	0.00
chr1_hap1	84167056	AluY	Sense	16	AAAAACTCACAAGTGG	57	0.00
chr1_hap1	135602742	AluY	Sense	17	ATAAAGCAGCAGAACTG	41	0.00
chr1_hap1	176727588	AluYa5	Sense	11	AAGAATGTTGA	24	0.04
chr2_hap1	170690878	AluYa5	Antisense	13	GACCTCATTTCTA	39	0.08
chr2_hap1	198786241	AluYa5	Antisense	18	GGAAAATCATTAACTTTA	42	0.00
chr3_hap1	36808900	AluYc	Antisense	11	GAAATCAGTCT	28	0.00
chr4_hap1	68633195	AluYa5	Antisense	14	CAACTTTGGTTTTT	99	0.00
chr4_hap1	104523183	AluYg6	Sense	15	GAAAATGCTAAAGGC	79	0.00
chr4_hap2	65226148	AluYa5	Sense	17	AAAAAGACACTAATTTA	79	0.00
chr6_hap1	47771164	AluYb8	Antisense	18	GATGACAGCTTAACTCTT	119	0.00
chr6_hap1	60438603	AluY	Antisense	12	GATAGAGCAGTT	42	0.14
chr6_hap1	91253973	AluY	Antisense	12	CACAAGCATTCT	34	0.00
chr6_hap1	91909369	AluYk2	Sense	9	AAGATTATA	57	0.00
chr6_hap1	103729153	AluYa5	Sense	14	AAAAAATATTGATC	80	0.00
chr6_hap1	170029052	AluYb8	Antisense	18	GGTAATCTTCCAGATTCT	31	0.00
chr6_hap2	76108804	AluYb8	Sense	16	AGAAAGCATGAAGCTG	73	0.00
chr6_hap2	148611276	AluYb8	Antisense	13	AGCACCATTTATT	40	0.18
chr7_hap2	90506402	AluYa5	Antisense	17	ATGTGCCACATTTTCTT	83	0.00
chr8_hap1	18412380	AluYb8	Antisense	17	AAAAGTTCTTTTTTTCT	79	0.00
chr8_hap1	135685705	AluYa5	Antisense	8	AACCTTCT	42	0.00
chr8_hap2	42738991	AluYa5	Antisense	10	TAAGGTAGCT	55	0.00
chr8_hap2	51723185	AluYk2	Sense	12	AAAGACACATGC	26	0.00
chr8_hap2	92608133	AluYb9	Sense	17	AAAAATATGGAAAGTCA	35	0.00
chr8_hap2	127839152	AluYa5	Sense	15	GAAAACTGGCACAAG	29	0.03
chr10_hap1	115958422	AluYa5	Antisense	16	CTGCCACTCCTCCTTT	64	0.00
chr10_hap2	9718078	AluYa5	Sense	12	GAAGAAGAGAGA	25	0.00
chr10_hap2	74978969	AluYa5	Sense	15	AAAGACTACAAATTG	50	0.00
chr11_hap1	137478201	AluY	Sense	11	GAAAACATACA	83	0.00
chr11_hap2	90051026	AluYb8	Antisense	15	GGGATTGGCTTTTAT	92	0.00
chr12_hap1	20298293	AluYa5	Antisense	15	TTATTTGTATTTTTT	55	0.00
chr12_hap2	85693478	AluYk2	Antisense	15	TTTTTTTTGCTTCTT	147	0.00
chr12_hap2	127435699	AluYk12	Antisense	9	CTGTTTCTT	28	0.07
chr12_hap2	128464422	AluYe5	Sense	14	AAAAGAAATAAAAG	59	0.00
chr13_hap1	36254334	AluYa5	Antisense	14	GAGACCCCATCTCT	38	0.00
chr13_hap2	82636190	AluYh7	Antisense	15	AATTTTTATTTTTTT	20	0.00
chr14_hap1	8559263	AluYb8	Sense	15	AAAAAGTCTGTTTCC	51	0.00

## Data Availability

Raw sequencing data will be deposited to SRA at the time of publication. The full analysis pipeline is available at https://github.com/sudmantlab/hifi_dn_alu. *nova* and *vesper* are individually available at https://github.com/sudmantlab/nova and https://github.com/sudmantlab/vesper.
