# Supplementary material for: Direct identification of de novo mobile element insertions from single molecule sequencing of human sperm
Source: bioRxiv. 2025 Oct 26:2025.10.25.684559. Preprint. [Version 1] doi: 10.1101/2025.10.25.684559 (PMC12633430; doi:10.1101/2025.10.25.684559)
Supplement: 1 [file NIHPP2025.10.25.684559V1-supplement-1.pdf]

bioRxiv preprint doi: https://doi.org/10.1101/2025.10.25.684559; this version posted October 26, 2025. The copyright holder for this preprint (which was not certified by peer review) is the author/funder, who has granted bioRxiv a license to display the preprint in perpetuity. It is made available under aCC-BY 4.0 International license.

Supplementary Tables

Supplementary Table 1: A table containing the full range of sequence features of individual Alu insertions.

Supplementary Table 2: A table of the upstream and downstream sequence contexts around the insertion breakpoints.

bioRxiv preprint doi: https://doi.org/10.1101/2025.10.25.684559; this version posted October 26, 2025. The copyright holder for this preprint (which was not certified by peer review) is the author/funder, who has granted bioRxiv a license to display the preprint in perpetuity. It is made available under aCC-BY 4.0 International license.

Supplementary Figures

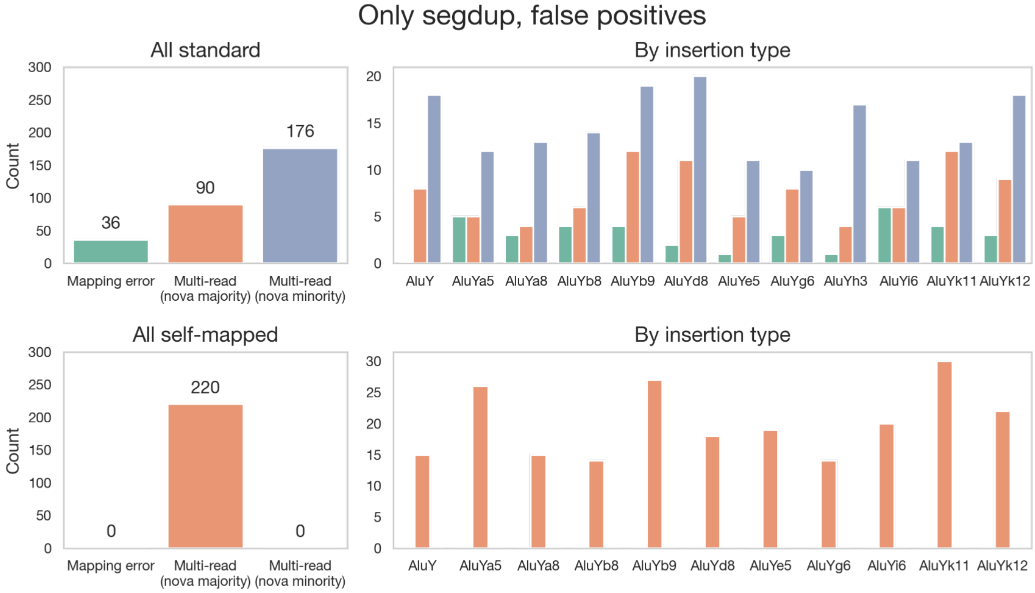

Supplementary Figure 1: False positive categorization in the segmental duplication targeted *nova* simulation.

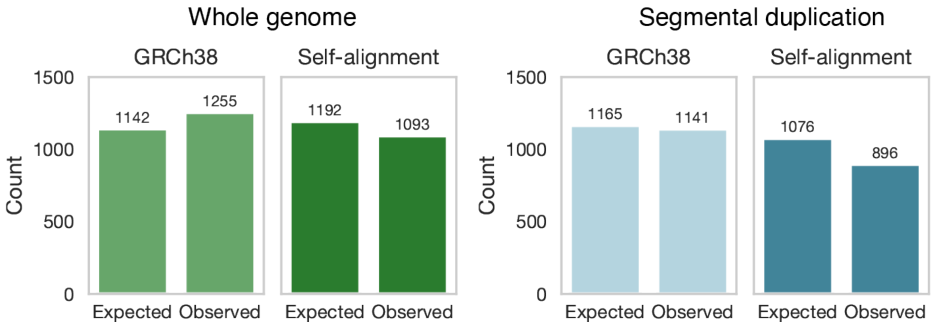

Supplementary Figure 2: Expected versus observed variant counts in each *nova* simulation. “Expected” refers to the number of synthetic single-read Alu insertions spiked into the baseline dataset. “Observed” refers to the number of reads identified in the output VCF.

bioRxiv preprint doi: https://doi.org/10.1101/2025.10.25.684559; this version posted October 26, 2025. The copyright holder for this preprint (which was not certified by peer review) is the author/funder, who has granted bioRxiv a license to display the preprint in perpetuity. It is made available under aCC-BY 4.0 International license.

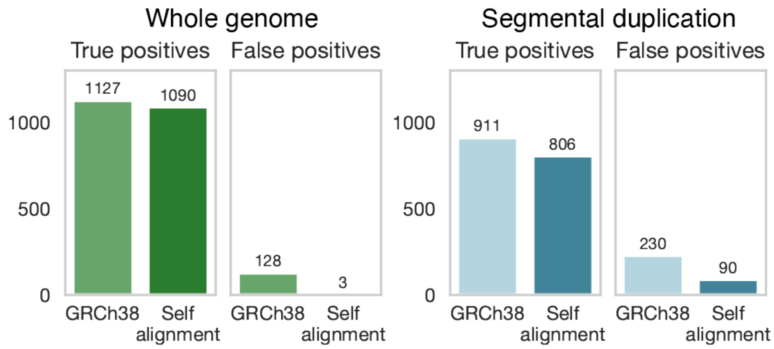

Supplementary Figure 3: True positive and false positive counts in each *nova* simulation.

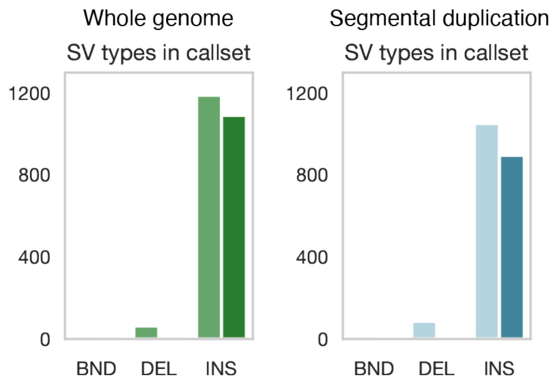

Supplementary Figure 4: Distribution of SV types called by *sniffles2* in each *nova* simulation.

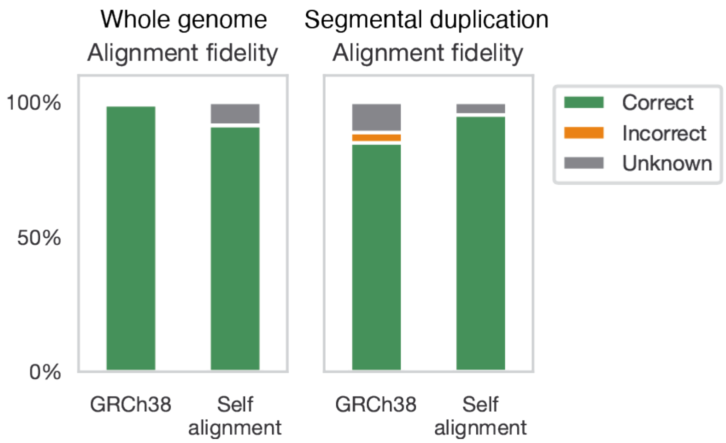

bioRxiv preprint doi: https://doi.org/10.1101/2025.10.25.684559; this version posted October 26, 2025. The copyright holder for this preprint (which was not certified by peer review) is the author/funder, who has granted bioRxiv a license to display the preprint in perpetuity. It is made available under aCC-BY 4.0 International license.

Supplementary Figure 5: Alignment fidelity of synthetic single-read Alu insertions in each *nova* simulation.

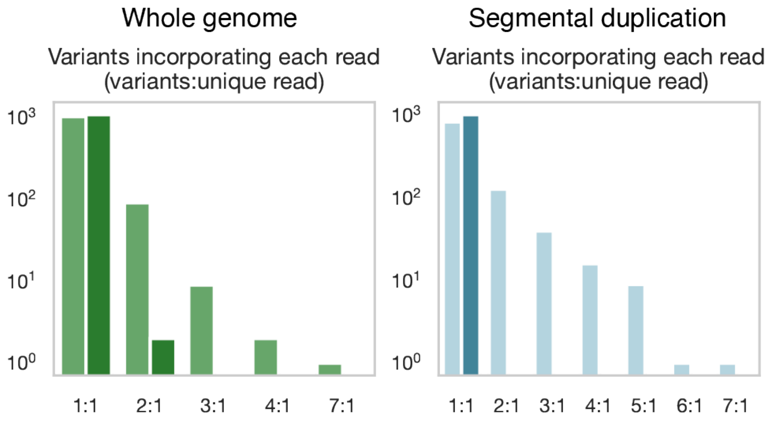

Supplementary Figure 7: Read reuse in each *nova* simulation. Each unique *nova* read should support one unique variant. Reads are considered "reused" if more than one variant is supported by the read.

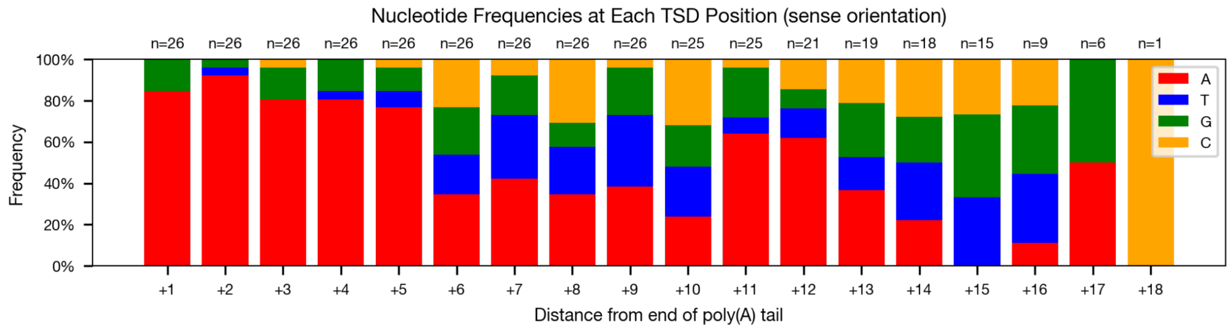

bioRxiv preprint doi: https://doi.org/10.1101/2025.10.25.684559; this version posted October 26, 2025. The copyright holder for this preprint (which was not certified by peer review) is the author/funder, who has granted bioRxiv a license to display the preprint in perpetuity. It is made available under aCC-BY 4.0 International license.

Supplementary Figure 7: Frequency of nucleotides in the target site sequence of sense-oriented Alu insertions, exhibiting proximal and distal A enrichment. The number of TSDs with at least X length is denoted above the bars.

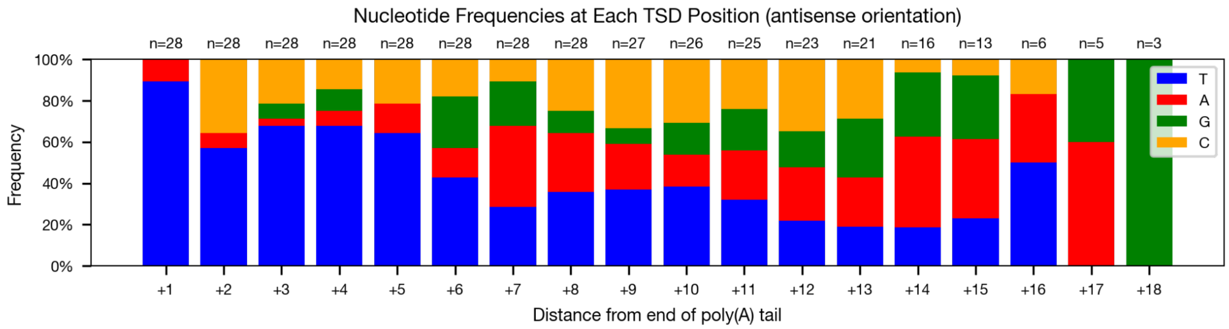

bioRxiv preprint doi: https://doi.org/10.1101/2025.10.25.684559; this version posted October 26, 2025. The copyright holder for this preprint (which was not certified by peer review) is the author/funder, who has granted bioRxiv a license to display the preprint in perpetuity. It is made available under aCC-BY 4.0 International license.

Supplementary Figure 8: Frequency of nucleotides in the target site sequence of antisense-oriented Alu insertions, exhibiting proximal and distal T enrichment. The number of TSDs with at least X length is denoted above the bars.

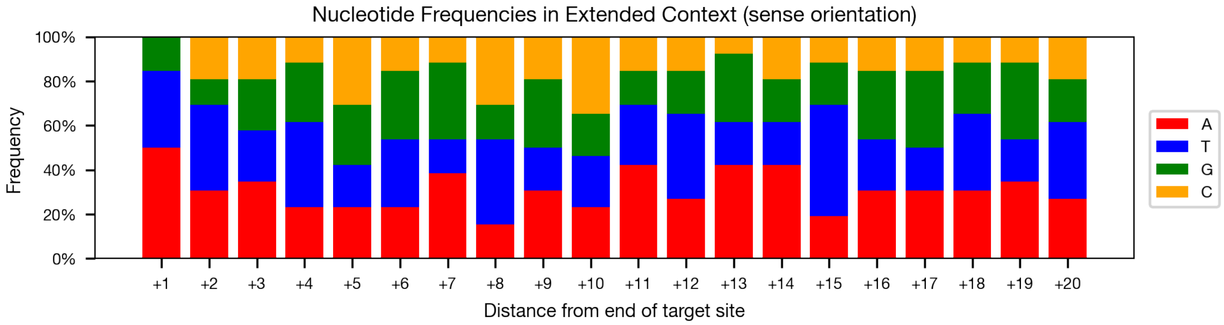

Supplementary Figure 9: Frequency of nucleotides in the 20 bp post-target site context of sense-oriented Alu insertions.

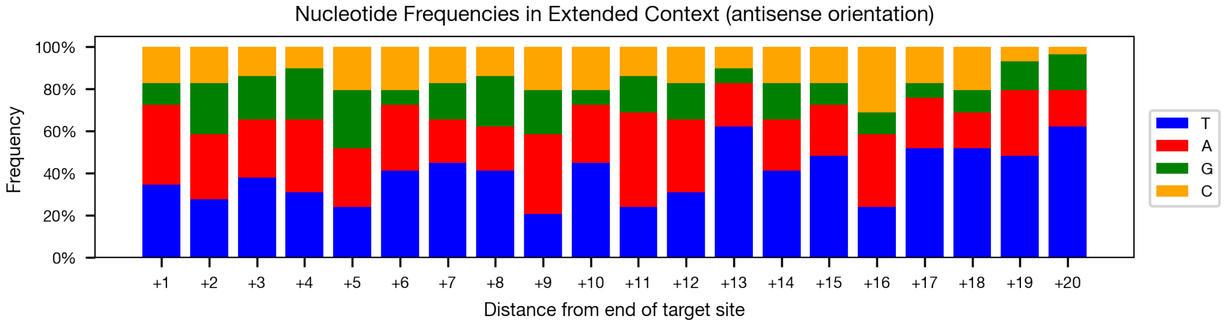

Supplementary Figure 10: Frequency of nucleotides in the 20 bp post-target site context of antisense-oriented Alu insertions.
